# NK cell HMGB1 mediates IL-2-induced “systemic autophagic” toxicity

**DOI:** 10.1186/2051-1426-1-S1-P123

**Published:** 2013-11-07

**Authors:** Guanqiao Li, Xiaoyan Liang, Michael Lotze

**Affiliations:** 1 Dept of Immunology, Univ of Pittsburgh Cancer Center, Pittsburgh, PA, USA

## Background and objectives

High dose Interleukin 2 (HDIL-2) treatment has durable antitumor effects in 5-10% of patients with metastatic melanoma and RCC. The application and efficacy of HDIL-2 treatment is limited due to substantial toxicity. We have previously shown that the toxicity results from cytokine-induce autophagy, which we term a “systemic autophagic syndrome”. Depletion of individual induced cytokines or B or T cells can’t rescue murine mortality with IL-2/IL-12 administration, but only NK cell depletion. Here, we hypothesize that the “systemic autophagic” toxicity is critically mediated by NK cells.

## Methods

Immunodeficient mice (RAG1-/- and NSG mice), hepatic metastases model, flow cytometry, ELISA, immunofluorescence and western blotting, transmission electron microscopy.

## Results

RAG1-/- mice containing an exaggerated NK cell population and function but no T or B cells die unlike wild type (WT) mice with HDIL-2 alone. Systemic autophagy was detected by autophagosome (TEM) and LC3-II (Immunofluorescence and western blot) in the lung, liver and kidney. Pre-treatment with the autophagy inhibitor chloroquine (CQ) mediated a modest protective effect, while NK elimination completely abrogates toxicity, associated with dramatically reduced serum HMGB1 and IL-6 levels. Adoptive transfer of WT B6 splenocytes with NK depletion can partially rescue the effects, with an enhanced population of CD3+CD4+CD25+ Tregs. Additionally, NSG mice adoptively transferred with RAG1-/- mice splenocytes display dose-dependent toxicity, assessed by relative lung weight and edema(H&E staining), serum HMGB1 and sRAGE (a novel indicator of toxicity and cancer prognosis) level and LC3 punctae formation. IL-2/18 combination which kills WT B6 mice, but NK elimination allows survival. NK cells generated from precursors show similar activated status but less mature phenotypes. Notably, anti-HMGB1 neutralizing antibodies can partly abrogate the severe syndrome of RAG mice, suggesting that HMGB1 may be essential to IL-2 toxicity. NKH mice lacking HMGB1 using floxed HMGB1 mice backcrossed into NKp46-cre mice specifically are viable, demonstrating that NK HMGB1 is required for systemic toxicity.

## Conclusions

IL-2 immunotherapy induces a “systemic autophagic” toxicity. The autophagy inhibitor, chloroquine, as well as administration of the HMGB1 neutralizing antibody, NK abolishment, or depletion of HMGB1 solely in NK cells promotes protection from IL-2 lethality. Thus, HMGB1 mediates the toxicity of IL-2 administration and possibly other immunotherapies.

